# Awareness of ovarian cancer risk and protective factors: A national cross-sectional study from Palestine

**DOI:** 10.1371/journal.pone.0265452

**Published:** 2022-03-21

**Authors:** Mohamedraed Elshami, Aya Tuffaha, Areej Yaseen, Mohammed Alser, Ibrahim Al-Slaibi, Hadeel Jabr, Sara Ubaiat, Salma Khader, Reem Khraishi, Inas Jaber, Zeina Abu Arafeh, Sondos Al-Madhoun, Aya Alqattaa, Asmaa Abd El Hadi, Ola Barhoush, Maysun Hijazy, Tamara Eleyan, Amany Alser, Amal Abu Hziema, Amany Shatat, Falasteen Almakhtoob, Balqees Mohamad, Walaa Farhat, Yasmeen Abuamra, Hanaa Mousa, Reem Adawi, Alaa Musallam, Nasser Abu-El-Noor, Bettina Bottcher

**Affiliations:** 1 Division of Surgical Oncology, Department of Surgery, University Hospitals Cleveland Medical Center, Cleveland, OH, United States of America; 2 Ministry of Health, Gaza, Palestine; 3 Faculty of Medicine, An-Najah National University, Nablus, Palestine; 4 Faculty of Medicine, Al-Quds University, Jerusalem, Palestine; 5 Almakassed Hospital, Jerusalem, Palestine; 6 Faculty of Medicine, Islamic University of Gaza, Gaza, Palestine; 7 Faculty of Medicine, Al-Quds University, Bethlehem, Palestine; 8 Faculty of Medicine, Al-Azhar university-Gaza, Gaza, Palestine; 9 Al-shiffa Hospital, Gaza, Palestine; 10 Faculty of Medicine, Palestine Polytechnic University, Hebron, Palestine; 11 Beit Jala Governmental Hospital, Bethlehem, Palestine; 12 Faculty of Medicine, Al-Quds University, Jenin, Palestine; 13 Al-Aqsa Hospital, Deir Albalah, Palestine; 14 Faculty of Nursing, Islamic University of Gaza, Gaza, Palestine; New York University Grossman School of Medicine, UNITED STATES

## Abstract

**Introduction:**

Having a good awareness of ovarian cancer (OC) risk and protective factors could facilitate early diagnosis. This study aimed to assess Palestinian women’s awareness about OC risk and protective factors and to identify the factors associated with having good awareness.

**Methods:**

A cross-sectional study was conducted from July 2019 to March 2020 in the two main areas of Palestine: the West Bank and Jerusalem (WBJ) and the Gaza Strip. A translated-into-Arabic version of the validated OC awareness measure was utilized. Adult women attending hospitals, primary healthcare centers, and public spaces at 11 governorates were invited to participate. The awareness level was categorized based on the number of factors recognized: poor (0 to 5), fair (6 to 10) and good (11 to 15).

**Results:**

Of the 6095 women approached, 5618 agreed and completed the questionnaire (response rate = 92.1%). The final analysis included 5411 questionnaires. The most identified modifiable OC risk factor was ‘being a smoker’ (n = 4024, 74.4%), whereas the least identified was ‘having in vitro fertilization treatment’ (n = 1652, 30.5%). The most identified non-modifiable OC risk factor was ‘having ovarian cysts’ (n = 3136, 58.0%), whereas the least identified was ‘having endometriosis’ (n = 1880, 34.7%). The most identified OC protective factor was ‘breastfeeding’ (n = 4770, 88.2%), whereas the least identified was ‘using the pill for a long time’ (n = 930, 17.2%). Only 820 women (15.2%) displayed good awareness of OC risk and protective factors. Women from the Gaza Strip were slightly more likely than women from the WBJ to have good awareness (16.4% vs. 14.2%). In contrast, post-secondary education, higher monthly income, being married, and knowing someone with cancer were associated with an increase in the likelihood of displaying good awareness.

**Conclusion:**

The overall awareness of OC risk and protective factors in this study was low. Educational interventions are needed to improve Palestinian women’s awareness.

## Introduction

Ovarian cancer (OC) is the second most deadly gynecological cancer worldwide with an estimated number of 207,252 deaths in 2020 [1]. After corpus uterine cancers, OC is the second most frequent gynecological cancer in Palestine [2]. The number of Palestinian women diagnosed with OC in 2020 was 74 women, of whom 53 (71.6%) died [2]. Low-resource settings, such as Palestine, necessitate finding efficient, cost-effective approaches to mitigate the high mortality of OC.

Risk factors of OC can be divided into modifiable and non-modifiable factors. Non-modifiable risk factors include family history of OC, older age, history of endometriosis or ovarian cysts, having no children, and early menarche [3–8]. Modifiable risk factors include smoking, obesity, having in vitro fertilization (IVF) treatment, and long-term use of hormonal replacement therapy [6, 9, 10]. On the other hand, protective factors of OC include breast feeding, using oral contraceptive pills and bilateral salpingo-oophorectomy [4, 5, 11, 12].

Promoting the awareness of OC risk and protective factors may lead to behavioral changes that could lower women’s risk to develop OC. These include smoking cessation, encouraging breastfeeding, having a lower body mass index, and decreasing or stopping the use of hormonal replacement therapy if not needed [4, 6, 13]. Raising the awareness of OC risk and protective factors could contribute to enhancing early diagnosis of OC [14]. This is especially critical for countries with under-funded healthcare systems, such as Palestine.

This national study aimed to: (i) assess Palestinian women’s awareness level of OC risk and protective factors, (ii) compare awareness levels between women living in the Gaza Strip vs. those living in the West Bank and Jerusalem (WBJ), and (iii) identify the factors associated with good awareness.

## Materials and methods

### Study design and population

This was a cross-sectional study conducted between July 2019 and March 2020 in the two main areas of Palestine: the Gaza Strip and the WBJ. Palestinian women aged 18 or over were the study population. Female visitors to Palestinian government hospitals, primary healthcare centers, and public spaces, such as malls, markets, gardens, restaurants, churches, mosques, and transportation stations, were recruited. Women with non-Palestinian citizenship, women working or studying in a health-related field, and those visiting oncology departments or clinics at the time of data collection were all excluded from the study.

### Sampling methods

Eligible women were recruited using a convenience sampling method from governmental hospitals, primary healthcare centers and public spaces in 11 governorates across Palestine. This was intended to make the study cohort resemble the Palestinian community by recruiting participants from different places and with varying sociodemographics [15–17].

### Measurement tool and data collection

A modified version of the OC Awareness Measure (OCAM) was utilized for data collection. The OCAM is a validated tool that was created to assess public awareness of OC [6]. Two bilingual experts first translated the original OCAM from English into Arabic, and then another two bilingual experts back-translated it into English. Five experts in the fields of gynecologic oncology, public health, and survey design reviewed the Arabic version of the OCAM for content validity and accuracy of translation. A pilot study (n = 128) was then conducted to test the clarity of the Arabic version of the OCAM. Participants included in the pilot study were excluded from the final analysis. Internal consistency of the Arabic OCAM was evaluated using Cronbach’s Alpha, which reached an acceptable value of 0.734.

For the purposes of this study, the same 12 OC risk factors mentioned in the original OCAM were used in the questionnaire. However, three OC protective factors that were not part of the original OCAM were added. Those included breastfeeding, the use of oral contraceptive pills, and undergoing bilateral salpingo-oophorectomy. Exploring the awareness of those factors in Palestine was deemed important due to common misconceptions about them [18], therefore, they were added. The final questionnaire consisted of three sections. The first section described the sociodemographics of study participants. The second section assessed the participant’s awareness of 12 OC risk factors. The third section assessed the participant’s awareness of three OC protective factors. The awareness of OC risk and protective factors was evaluated using a 5-point Likert scale (1 = strongly disagree, 5 = strongly agree).

The electronic tool ‘Kobo Toolbox’ was utilized to collect data [19]. Kobo Toolbox is a secure tool that can be used both offline and online on mobile devices. Participants were invited to fill out the questionnaire in a face-to-face interview. Female data collectors with medical backgrounds were trained on how to use Kobo Toolbox, how to approach the study participants, and how to facilitate their completion of the questionnaire.

### Ethical considerations

Ethical approval was obtained from the Palestinian Ministry of Health to collect data from hospitals and primary healthcare centers. The study was also approved by the Ethics Committee at the Islamic University of Gaza and the Helsinki Committee for Research Ethics in the Gaza Strip. The purpose of the study was explained to study participants and a written informed consent was taken before enrollment into the study. Data were collected anonymously, and confidentiality was kept throughout the study.

### Statistical analysis

Characteristics of participants were summarized utilizing descriptive statistics. The median and interquartile range (IQR) were used to describe continuous non-normally distributed variables. Frequencies (n) and percentages (%) were utilized to describe categorical variables. In order to reflect the age-related risk of OC, age was categorized into two groups: 18 to 44 years and 45 years or older (at-risk group) [6]. The onset of menstruation (menarche) was categorized into three categories; early (≤ 10 years), normal (11–15 years), and late (≥ 16 years) [20]. The cutoff of 1450 NIS (about $450) was selected to categorize the monthly income into two categories since it was the minimum wage in Palestine [21]. A baseline comparison between characteristics of participants living in the Gaza Strip and of those living in the WBJ was performed using the Kruskal-Wallis test if the variable was continuous or the Pearson’s Chi-square test if it was categorical.

Answers to questions about OC risk and protective factors with ‘strongly agree’ or ‘agree’ were considered correct, whereas answers with ‘strongly disagree’, ‘disagree’, or ‘not sure’ were considered incorrect. OC risk factors were categorized into two categories: modifiable and non-modifiable. Identifying each of the OC risk/protective factors was described utilizing frequencies and percentages with comparisons performed utilizing Pearson’s Chi-Square test. This was followed by running bivariable and multivariable logistic regression. The model of the multivariable analysis included age-group, menarche, educational level, occupation, monthly income, residency, having a chronic disease, knowing someone with cancer, marital status, and site of data collection. This model was determined a priori based on previous studies [14, 16, 22–25]. Results of bivariable analyses are provided in the supplementary materials.

To evaluate the participant’s awareness level about OC risk and protective factors, a scoring system was utilized. Similar scoring systems were used in previous studies [15–17, 26, 27]. The total score (ranging from 0 to 15) was calculated and categorized into three categories: poor (0 to 5), fair (6 to 10) and good awareness (11 to 15). Pearson’s Chi-Square test was used to compare the awareness level between the participants from the Gaza Strip vs. the WBJ. The association between participant characteristics and having good awareness level was tested using bivariable and multivariable logistic regression analyses.

Missing data were hypothesized to be missed completely at random. Therefore, complete case analysis was used to handle missing data, where questionnaires with at least one variable missing were excluded from the analysis. Data were analyzed using Stata software version 16.0 (StataCorp, College Station, Texas, United States).

## Results

### Participant characteristics

Of 6095 participants approached, 5618 agreed and completed the questionnaire (response rate = 92.1%). A total of 5411 questionnaires were included in the final analysis (158 had missing data and 49 were ineligible): 3133 from the WBJ and 2278 from the Gaza Strip. The median age [IQR] for all study participants was 32.0 years [24.0, 44.0] (Table 1). Participants living in the Gaza Strip were more likely to be younger, have lower education and monthly income, and suffer from less chronic diseases than participants living in the WBJ.

**Table 1:** Characteristics of study participants.

Characteristic	Total	Gaza Strip	WBJ	p-value
	(n = 5411)	(n = 2278)	(n = 3133)	
**Age,** median [IQR]	32 [24, 44]	30 [24, 40]	34 [25, 46]	<0.001
**Age group,** n (%)				
18 to 44	4151 (76.7)	1872 (82.2)	2279 (72.2)	<0.001
45 or older	1260 (23.3)	406 (17.8)	854 (27.3)	
**Menarche,** n (%)				
Early (≤ 10 years)	65 (1.2)	20 (0.9)	45 (1.4)	<0.001
Normal (11–15 years)	4658 (86.1)	1923 (84.4)	2735 (87.3)	
Late (≥ 16 years)	688 (12.7)	335 (14.7)	353 (11.3)	
**Educational level,** n (%)				
Secondary or below	3016 (55.7)	1330 (58.4)	1686 (53.8)	<0.001
Post-secondary	2395 (44.3)	948 (41.6)	1447 (46.2)	
**Occupation,** n (%)				
Unemployed/housewife	3671 (67.8)	1837 (80.6)	1834 (58.5)	<0.001
Employed	1095 (20.2)	254 (11.2)	841 (26.8)	
Retired	47 (0.9)	9 (0.4)	38 (1.2)	
Student	598 (11.1)	178 (7.8)	420 (13.5)	
**Monthly income ≥ 1450 NIS,** n (%)	3330 (61.5)	474 (20.8)	2856 (91.2)	<0.001
**Having a chronic disease,** n (%)	1097 (20.3)	350 (15.4)	747 (23.8)	<0.001
**Knowing someone with cancer,** n (%)	2746 (50.8)	1104 (48.5)	1642 (52.4)	0.016
**Marital status,** n (%)				
Single	1248 (23.1)	374 (16.4)	874 (27.9)	<0.001
Married	3952 (73.0)	1836 (80.6)	2116 (67.5)	
Divorced	88 (1.6)	33 (1.4)	55 (1.8)	
Widowed	123 (2.3)	35 (1.6)	88 (2.8)	
**Site of data collection,** n (%)				
Public Spaces	1645 (30.4)	596 (26.2)	1049 (33.5)	<0.001
Hospitals	1735 (32.1)	650 (28.5)	1085 (34.6)	
Primary healthcare centers	2031 (37.5)	1032 (45.3)	999 (31.9)	

n = number of participants, IQR = interquartile range, WBJ = West Bank and Jerusalem.

### Identifying OC risk and protective factors

The most identified modifiable risk factor was ‘being a smoker’ (n = 4024, 74.4%), whereas the least identified was ‘having IVF treatment’ (n = 1652, 30.5%) (Table 2). The most identified non-modifiable risk factor was ‘having ovarian cysts’ (n = 3136, 58.0%), whereas the least identified was ‘having endometriosis’ (n = 1880, 34.7%). On the other hand, the most identified protective factor was ‘breastfeeding’ (n = 4770, 88.2%), whereas the least identified factor was ‘using the pill for a long time’ (n = 930, 17.2%).

**Table 2:** Recognition of ovarian cancer risk and protective factors.

Factor	Total	Gaza Strip	WBJ	p-value
	(n = 5411)	(n = 2278)	(n = 3133)	
	n (%)	n (%)	n (%)	
**Modifiable risk factors**				
Being a smoker	4024 (74.4)	1777 (78.0)	2247 (71.7)	<0.001
Using hormone replacement therapy	3053 (56.4)	1272 (55.8)	1781 (56.8)	0.46
Being overweight	2453 (45.3)	1116 (49.0)	1337 (42.7)	<0.001
Using talcum powder in the genital area	2365 (43.7)	973 (42.7)	1392 (44.4)	0.21
Having IVF treatment	1652 (30.5)	780 (34.2)	872 (27.8)	<0.001
**Non-modifiable risk factors**				
Having ovarian cysts	3136 (58.0)	1342 (58.9)	1794 (57.3)	0.22
Being over 50 years old	2994 (55.3)	1332 (58.5)	1662 (53.0)	<0.001
Having a close relative with ovarian cancer	2980 (55.1)	1120 (49.2)	1860 (59.4)	<0.001
Having gone through the menopause	2943 (54.4)	1335 (58.6)	1608 (51.3)	<0.001
Having a history of breast cancer	2693 (49.8)	1183 (51.9)	1510 (48.2)	0.007
Having no children	1926 (35.6)	953 (41.8)	973 (31.1)	<0.001
Having endometriosis	1880 (34.7)	855 (37.5)	1025 (32.7)	<0.001
**Protective factors**				
Breastfeeding	4770 (88.2)	2092 (91.8)	2678 (85.5)	<0.001
Undergoing prophylactic bilateral oophorectomy	2127 (39.3)	798 (35.0)	1329 (42.4)	<0.001
Using the pill for a long time	930 (17.2)	417 (18.3)	513 (16.4)	0.06

n = number of participants. WBJ = West Bank and Jerusalem, IVF = in vitro fertilization.

### Good awareness and its associated factors

Only 820 participants (15.2%) displayed good awareness of OC risk and protective factors (Table 3). Participants from the Gaza Strip were slightly more likely than participants from the WBJ to have a good level of awareness (16.4% vs. 14.2%). On the multivariable analysis, living in the WBJ and visiting primary healthcare centers were both associated with a decrease in the likelihood of having a good awareness level of OC risk and protective factors (Table 4). On the other hand, post-secondary education, high monthly income (≥1450 NIS), being married, and knowing someone with cancer were all associated with an increase in the likelihood of displaying a good awareness level.

**Table 3:** Awareness level of ovarian cancer risk and protective factors among study participants.

Level	Total	Gaza Strip	WBJ	p-value
	n (%)	n (%)	n (%)	
Poor	1445 (26.7)	522 (22.9)	923 (29.5)	<0.001
Fair	3146 (58.1)	1382 (60.7)	1764 (56.3)	
Good	820 (15.2)	374 (16.4)	446 (14.2)	

n = number of participants, WBJ = West Bank and Jerusalem.

**Table 4:** Association of participant characteristics with having a good awareness of ovarian cancer risk and protective factors.

Characteristic	Good awareness			
	COR (95% CI)	P	AOR (95% CI)*	P
**Age group**				
18 to 44	Ref	Ref	Ref	Ref
45 or older	1.16 (0.98–1.38)	0.09	1.17 (0.96–1.43)	0.12
**Menarche**				
Normal	Ref	Ref	Ref	Ref
Early	1.71 (0.96–3.06)	0.07	1.49 (0.83–2.70)	0.19
Late	1.09 (0.87–1.35)	0.47	1.07 (0.86–1.34)	0.56
**Educational level**				
Secondary or below	Ref	Ref	Ref	Ref
Post-secondary	1.23 (1.06–1.43)	0.006	1.46 (1.23–1.74)	<0.001
**Occupation**				
Unemployed/housewife	Ref	Ref	Ref	Ref
Employed	1.00 (0.83–1.20)	0.96	0.96 (0.77–1.19)	0.71
Retired	0.64 (0.25–1.64)	0.36	0.56 (0.21–1.46)	0.23
Student	0.75 (0.58–0.98)	0.033	0.91 (0.65–1.26)	0.56
**Monthly income**				
< 1450 NIS	Ref	Ref	Ref	Ref
≥ 1450 NIS	1.04 (0.89–1.21)	0.62	1.25 (1.00–1.57)	0.048
**Residency**				
Gaza Strip	Ref	Ref	Ref	Ref
WBJ	0.85 (0.73–0.98)	0.027	0.69 (0.55–0.85)	0.001
**Having a chronic disease**				
No	Ref	Ref	Ref	Ref
Yes	1.01 (0.84–1.21)	0.94	0.95 (0.75–1.17)	0.65
**Knowing someone with cancer**				
No	Ref	Ref	Ref	Ref
Yes	1.36 (1.17–1.58)	<0.001	1.30 (1.11–1.51)	0.001
**Marital status**				
Single	Ref	Ref	Ref	Ref
Married	1.42 (1.17–1.72)	<0.001	1.53 (1.20–1.94)	<0.001
Divorced	0.73 (0.35–1.54)	0.41	0.76 (0.35–1.62)	0.47
Widowed	1.42 (0.85–2.36)	0.18	1.48 (0.85–2.58)	0.16
**Site of data collection**				
Public Spaces	Ref	Ref	Ref	Ref
Hospitals	0.98 (0.82–1.18)	0.87	0.96 (0.80–1.16)	0.71
Primary healthcare centers	0.60 (0.50–0.72)	<0.001	0.56 (0.46–0.68)	<0.001

COR = crude odds ratio, AOR = adjusted odds ratio, CI = confidence interval, WBJ = West Bank and Jerusalem.

* Adjusted for age-group, menarche, educational level, occupation, monthly income, marital status, residency, having a chronic disease, knowing someone with cancer, and site of data collection.

### Association between identifying OC modifiable risk factors and participant characteristics

On the multivariable analysis, participants form the WBJ were less likely than the participants from the Gaza Strip to identify all modifiable risk factors except ‘using talcum powder in the genital area’, where no difference was noticed (S1 Table in S1 File). On the contrary, married women were more likely than single women to identify all modifiable risk factors except ‘having IVF treatment’ for which no difference was noticed.

### Association between identifying OC non-modifiable risk factors and participant characteristics

The multivariable analysis showed that women living in the WBJ were less likely than women living in the Gaza Strip to recognize all non-modifiable risk factors except ‘having a close relative with OC’ for which the opposite was noticed (S2 Table in S1 File). In addition, visitors to primary healthcare centers were less likely than visitors to public spaces to identify all non-modifiable risk factors except ‘having a close relative with OC’, where no difference was found. Conversely, participants who knew someone with cancer had a higher likelihood than those who did not to identify all non-modifiable risk factors.

### Association between identifying OC protective factors and participant characteristics

Women who knew someone with cancer were more likely to identify ‘breastfeeding’ and ‘undergoing prophylactic bilateral oophorectomy’ as OC protective factors (S3 Table in S1 File). On the other hand, women who were employed or recruited from primary healthcare centers were less likely to identify ‘breastfeeding’ and ‘undergoing prophylactic bilateral oophorectomy’ as protective factors.

## Discussion

The overall awareness of Palestinian women about OC risk and protective factors was found to be poor with only 15.2% displaying good awareness. Participants from the Gaza Strip were slightly more likely to show higher awareness levels than participants from the WBJ. The factors associated with having good awareness were post-secondary education, high monthly income(≥ 1450 NIS), being married, and knowing someone with cancer. The most identified modifiable risk factor was ’being a smoker’ while the least identified was ‘having IVF treatment’. The most identified non-modifiable risk factor was ‘having ovarian cysts’ while the least identified was ‘having endometriosis’. On the other hand, the most identified protective factor was ‘breastfeeding’ while the least identified was ‘using the pill for a long time’.

There has been a growing interest in measuring the awareness of OC and efforts are being made to promote women’s recognition of OC risk and protective factors [6, 14, 22, 24, 25, 28–31]. The Every Woman Study found that women who described their awareness of OC as ’good’ before having the diagnosis of OC were more likely to see a doctor within three months of the onset of symptoms and being diagnosed within one month of that visit [31]. Displaying a good awareness of OC risk and protective factors may help women to identify themselves as high-risk, hence, there is a greater chance to seek medical advice if they experience possible OC symptoms [6, 14]. In addition, recognition of OC modifiable risk factors as well as protective factors may lead to behavioral changes that could lower the risk of women to develop OC [32].

### Awareness level of OC risk and protective factors

In line with previous studies, this study showed poor awareness of OC risk and protective factors [23, 25, 29]. Mohamed and colleagues conducted a quasi-experimental study in Egypt and demonstrated an increase in the awareness of OC risk and protective factors after an interactive session that discussed information about OC [29]. The authors also found that the improvement in awareness remained after a three-month follow-up. Similarly, Puckett and colleagues reported an increase of OC awareness and confidence of women and healthcare providers to talk to each other about gynecologic cancer [22]. Such educational interventions could be effective in low- and middle-income countries, such as Palestine, where no awareness campaigns have taken place on OC so far. Nonetheless, creating awareness campaigns requires continuous funding and efforts, which can be challenging for low- and middle-income countries like Palestine. However, in the long-term, such campaigns could be a cost-effective investment, contribute to early diagnosis and, thus, decrease disease burden and improve outcomes [6, 14, 16, 31].

### Factors associated with good awareness level

In this study, participants with low education (secondary or below) were less likely to have good awareness of OC risk and protective factors. This opens the option of targeting educational programs to women with low levels of education by enhancing the existing curricula with more health-related topics including OC-related content in school curricula [16, 26, 27, 33, 34]. These interventions should be available to different groups in schools and freely introduced [35]. Repetitive exposure to educational interventions throughout women’s lifetimes might support reinforcement of information and help women form their own health literacy. Interestingly, women recruited from primary healthcare centers were less likely than women recruited from public spaces to display good awareness of OC risk and protective factors. Our research group previously found no association between visiting primary healthcare centers and having good awareness of OC symptoms [16]. A possible explanation for these findings could be that women visiting primary healthcare centers do not spend their waiting times to educate themselves by reading the available educational brochures, for example. Rather, women may prefer to interact with their treating physicians and solely discuss their presenting symptom, which could be unrelated to OC. This also may explain the finding that employed women, who have less time to read about health-related topics, had a lower likelihood to recognize some OC risk and protective factors than unemployed women.

On the other hand, married women and those who knew someone with cancer were more likely to display good awareness. Married women might be exposed to more health information during their visits to healthcare clinics for reproductive health. The repetitive exposure to such information might have helped women to develop better awareness of health-related topics including OC risk and protective factors [15–17]. Knowing someone with cancer may also encourage women to read and seek more information about health topics.

In this study, participants living in the Gaza Strip were more likely to have a good awareness of OC risk and protective factors than participants living in the WBJ. The political situation in Palestine, especially in the WBJ, and the security checkpoints between cities and sometimes within the same city, limit women’s movement and impede their access to healthcare services. This may in turn prevent women in the WBJ from shaping their health literacy. Another contributing factor to the observed difference in awareness between women in the Gaza Strip vs. the WBJ could be the impact of the United Nations Relief and Works Agency (UNRWA). The UNRWA plays a vital role in raising awareness about different health topics including cancer [36]. The UNRWA is a UN agency that aims to promote the relief and human development of Palestinian refugees. The number of refugees in the Gaza Strip is nearly 1.4 million comprising about 70% of the Gaza Strip population compared with a lower number of refugees in the WBJ of 871,537, which is about 28% of the WBJ population [37–39]. This may provide more opportunities to women in the Gaza Strip to come in contact with the various initiatives run by the UNRWA to raise health awareness. In 2011, the UNRWA established a Family Health Team approach, which included health education. These interventions had good impact on the awareness level [40]. Furthermore, the United Nations Children’s Fund (UNICEF) provided health education on breastfeeding, which is reflected in the relatively good awareness of breastfeeding as a protective factor for OC [41].

Therefore, educational interventions have the potential to allow women to form and develop their health literacy, including knowledge on OC risk and protective factors. This is especially significant in low- and middle-income countries, where 70% of global cancer mortality occurs [42]. A significant contributor to disease burden and mortality in low- and middle-income countries is late presentation for diagnosis and treatment [43, 44]. Difficulties to access treatment, financial burden of medical care and negative beliefs associated with a cancer diagnosis might all contribute to a delay in diagnosis [18, 27, 43, 45–47]. Negative beliefs may be fueled by shared local experiences as well as poor knowledge of treatment options and outcomes [18, 45, 48]. Therefore, improving knowledge and awareness of risk and protective factors might support healthy life choices and may have positive impact also on negative beliefs around cancer. This may be especially useful in low- and middle-income countries and contribute to a reduction of disease burden [18, 27, 43, 45].

### Future directions

Educational interventions are needed to raise the awareness of OC risk and protective factors in Palestine, which is an example of countries with low-resource settings. These should use channels that are accessible to locals (e.g., social media). Another option to deliver information could be through women’s visits to healthcare facilities. This will require training of healthcare professionals to facilitate the provision of information in order to improve public health literacy. OC is most common in older postmenopausal women [49]. Reaching this group may not be feasible through social media because of the lower utilization [50]. However, these women could be targeted by local media or text messages. The latter could be possible with the widespread availability of cellular phones across low- and middle-income countries such as Palestine [51].

### Strengths and limitations

The main strengths of this study include the large sample size, the high response rate, and the wide geographical coverage across Palestine. In addition, data were collected by face-to-face interviews minimizing the possibility that women may recheck their answers from the internet or other sources. On the other hand, limitations of this study include the convenience sampling, which may not guarantee the generation of a representative sample and might affect the generalizability of the results. However, the large sample size, high response rate, and the recruitment from different places across Palestine may mitigate this issue. Another limitation could be the smaller proportion of women at the highest risk of OC that were included in the study (i.e., women aged 65 and older). However, the Palestinian population is a young and growing population. Women ≥ 65 years make up only around 3% and women ≥ 45 years make up around 25% of the total female population [52], which corresponds to the percentage in the study sample.

## Conclusions

The awareness level of OC risk and protective factors among women included in this study was low with only 15.2% demonstrating good awareness. Women from the Gaza Strip were slightly more likely to demonstrate higher awareness than women from the WBJ. Higher education and monthly income, being married, and knowing someone with cancer were all associated with higher likelihood of having good awareness. The most commonly identified modifiable OC risk factor was ’being a smoker’ while the least identified was ‘having IVF treatment’. The most commonly identified non-modifiable OC risk factor was ‘having ovarian cysts’ while the least identified was ‘having endometriosis’. Finally, the most frequently identified OC protective factor was ‘breastfeeding’ while the least frequently identified protective factor was ‘using the pill for a long time’. The findings of this study open new opportunities for targeted educational interventions to facilitate prevention and early detection of OC.

## Supporting information

S1 File (DOC)
