# Supplementary material for: Awareness of ovarian cancer risk and protective factors: A national cross-sectional study from Palestine
Source: PLoS One. 2022 Mar 21;17(3):e0265452. doi: 10.1371/journal.pone.0265452 (PMC8936444; doi:10.1371/journal.pone.0265452)
Supplement: S1 File — (DOC) [file pone.0265452.s001.DOC]

**Awareness of Ovarian Cancer Risk and Protective Factors: A National Cross-sectional Study from Palestine**

Mohamedraed Elshami, MD, MMSc^1,2^*, Aya Tuffaha^3^*, Areej Yaseen^4^*, Mohammed Alser, MD^2^, Ibrahim Al-Slaibi, MD^5^, Hadeel Jabr, MD^6^, Sara Ubaiat^7^, Salma Khader^4^, Reem Khraishi^3^, Inas Jaber^4^, Zeina Abu Arafeh^4^, Sondos Al-Madhoun^8^, Aya Alqattaa^6^, Asmaa Abd El Hadi^6^, Ola Barhoush^4^, Maysun Hijazy^6^, Tamara Eleyan^4^, Amany Alser^9^, Amal Abu Hziema^6^, Amany Shatat^6^, Falasteen Almakhtoob^10^, Balqees Mohamad, MD^11^, Walaa Farhat^12^, Yasmeen Abuamra^8^, Hanaa Mousa^6^, Reem Adawi^4^, Alaa Musallam, MD^13^, Nasser Abu-El-Noor, PhD^14#^, Bettina Bottcher, MD, PhD^6#^

*Contributed equally as a first co-author.

^#^Contributed equally as a senior co-author.

^1^Division of Surgical Oncology, Department of Surgery, University Hospitals Cleveland Medical Center, Cleveland, OH, USA.
^2^Ministry of Health, Gaza, Palestine.
^3^Faculty of Medicine, An-Najah National University, Nablus, Palestine
^4^Faculty of Medicine, Al-Quds University, Jerusalem, Palestine.
^5^Almakassed Hospital, Jerusalem, Palestine. ^6^Faculty of Medicine, Islamic University of Gaza, Gaza, Palestine. ^7^Faculty of Medicine, Al-Quds University, Bethlehem, Palestine. ^8^Faculty of Medicine, Al-Azhar university-Gaza, Gaza, Palestine.
^9^Al-shiffa Hospital, Gaza, Palestine.
^10^Faculty of Medicine, Palestine Polytechnic University, Hebron, Palestine.
^11^Beit Jala Governmental Hospital, Bethlehem, Palestine.
^12^Faculty of Medicine, Al-Quds University, Jenin, Palestine. ^13^Al-Aqsa Hospital, Deir Albalah, Palestine. ^14^Faculty of Nursing, Islamic University of Gaza, Gaza, Palestine.

**Corresponding author**

Mohamedraed Elshami, MD, MMSc

Division of Surgical Oncology

Department of Surgery

University Hospitals Cleveland Medical Center

11100 Euclid Avenue, Lakeside 7100

Cleveland, OH 44106
Phone: 832-245-6055

Email: mohamedraed.elshami@gmail.com

| Characteristic | Being a smoker | | Using hormone replacement therapy | | Being overweight | | Using talcum powder in the genital area | | Having IVF treatment | |
| --- | --- | --- | --- | --- | --- | --- | --- | --- | --- | --- |
|  | **AOR (95% CI)*** | **p-value** | **AOR (95% CI)*** | **p-value** | **AOR (95% CI)*** | **p-value** | **AOR (95% CI)*** | **p-value** | **AOR (95% CI)*** | **p-value** |
| Age group  18to 44  45 or older | Ref  0.97 (0.82- 1.16) | Ref  0.75 | Ref  1.08 (0.93- 1.26) | Ref  0.30 | Ref  1.04 (0.90- 1.21) | Ref  0.57 | Ref  0.86 (0.74- 1.00) | Ref  0.051 | Ref  1.03 (0.87- 1.21) | Ref  0.73 |
| Menarche  Normal  Early  Late | Ref  1.07 (0.59- 1.93)  1.09 (0.90- 1.32) | Ref  0.82  0.39 | Ref  1.07 (0.64- 1.77)  0.93 (0.79- 1.10) | Ref  0.80  0.40 | Ref  1.04 (0.63- 1.70)  1.17 (0.99- 1.37) | Ref  0.88  0.06 | Ref  0.95 (0.58- 1.57)  0.92 (0.78- 1.08) | Ref  0.84  0.31 | Ref  1.16 (0.68- 1.95)  1.10 (0.93- 1.31) | Ref  0.59  0.26 |
| Educational level  Secondary or below  Post-secondary | Ref  0.91 (0.79- 1.05) | Ref  0.21 | Ref  1.46 (1.29- 1.66) | Ref  <0.001 | Ref  1.02 (0.90- 1.15) | Ref  0.77 | Ref  1.22 (1.07- 1.38) | Ref  0.002 | Ref  1.19 (1.04- 1.36) | Ref  0.01 |
| Occupation  Unemployed/housewife  Employed  Retired  Student | Ref  0.74 (0.62- 0.89)  0.89 (0.46- 1.74)  1.08 (0.84- 1.38) | Ref  0.001  0.74  0.55 | Ref  0.92 (0.78- 1.08)  1.32 (0.70- 2.50)  0.96 (0.77- 1.20) | Ref  0.32  0.40  0.74 | Ref  1.07 (0.91- 1.25)  0.83 (0.45- 1.51)  1.06 (0.84- 1.32) | Ref  0.43  0.54  0.64 | Ref  0.86 (0.73- 1.01)  0.80 (0.44- 1.47)  0.96 (0.77- 1.20) | Ref  0.07  0.47  0.70 | Ref  1.10 (0.93- 1.31)  0.78 (0.39- 1.58)  1.17 (0.93- 1.48) | Ref  0.27  0.50  0.19 |
| Monthly income  < 1450 NIS  ≥ 1450 NIS | Ref  1.06 (0.87- 1.28) | Ref  0.57 | Ref  1.49 (1.26- 1.76) | Ref  <0.001 | Ref  0.97 (0.82- 1.14) | Ref  0.70 | Ref  1.22 (1.04- 1.44) | Ref  0.017 | Ref  0.88 (0.74- 1.05) | Ref  0.16 |
| Residency  Gaza Strip  WBJ | Ref  0.71 (0.59- 0.86) | Ref  <0.001 | Ref  0.78 (1.26- 1.76) | Ref  0.003 | Ref  0.80 (0.68- 0.94) | Ref  0.006 | Ref  0.98 (0.83- 1.15) | Ref  0.78 | Ref  0.77 (0.65- 0.92) | Ref  0.003 |
| Having a chronic disease  No  Yes | Ref  0.96 (0.80- 1.14) | Ref  0.64 | Ref  1.04 (0.89- 1.21) | Ref  0.61 | Ref  1.02 (0.88- 1.19) | Ref  0.79 | Ref  1.09 (0.94- 1.27) | Ref  0.24 | Ref  0.95 (0.80- 1.12) | Ref  0.54 |
| Knowing someone with cancer  No  Yes | Ref  1.32 (1.17- 1.50) | Ref  <0.001 | Ref  1.07 (0.96- 1.19) | Ref  0.25 | Ref  1.08 (0.97- 1.21) | Ref  0.15 | Ref  1.09 (0.98- 1.22) | Ref  0.11 | Ref  1.03 (0.92- 1.16) | Ref  0.59 |
| Marital status  Single  Married  Divorced  Widowed | Ref  1.36 (1.14- 1.63)  2.34 (1.33- 4.14)  2.14 (1.28- 3.59) | Ref  0.001  0.003  0.004 | Ref  1.34 (1.14- 1.58)  0.93 (0.60- 1.45)  1.11 (0.74- 1.66) | Ref  <0.001  0.74  0.61 | Ref  1.20 (1.01- 1.41)  1.36 (0.87- 2.12)  1.10 (0.74- 1.65) | Ref  0.033  0.18  0.64 | Ref  1.27 (1.08- 1.50)  1.13 (0.72- 1.77)  1.41 (0.94- 2.10) | Ref  0.005  0.61  0.10 | Ref  1.08 (0.91- 1.29)  0.65 (0.38- 1.09)  1.22 (0.79- 1.88) | Ref  0.37  0.10  0.37 |
| Site of data collection  Public spaces  Hospitals  Primary healthcare centers | Ref  1.52 (1.28- 1.81)  0.87 (0.75- 1.02) | Ref  <0.001  0.08 | Ref  1.10 (0.95- 1.28)  0.85 (0.74- 0.98) | Ref  0.19  0.027 | Ref  1.26 (1.09- 1.46)  1.15 (1.00- 1.32) | Ref  0.001  0.044 | Ref  1.15 (0.99- 1.32)  1.08 (0.94- 1.25) | Ref  0.06  0.26 | Ref  0.71 (0.61- 0.83)  0.71 (0.61- 0.82) | Ref  <0.001  <0.001 |

AOR= adjusted odds ratio, CI= confidence interval, WBJ= West Bank and Jerusalem, IVF= in vitro fertilization.
* Adjusted for age-group, menarche, educational level, occupation, monthly income, residency, having a chronic disease, knowing someone with cancer, and site of data collection.

Supplementary table 1: Multivariable logistic regression analyzing the association between recognizing the modifiable risk factors of ovarian cancer and participant characteristics.

| Characteristic | Having ovarian cysts | | Being over 50 years old | | Having a close relative with ovarian cancer | | Having gone through the menopause | | Having a history of breast cancer | | Having no children | | Having endometriosis | |
| --- | --- | --- | --- | --- | --- | --- | --- | --- | --- | --- | --- | --- | --- | --- |
|  | **AO** **R (95% CI)*** | **p-value** | **AOR (95% CI)*** | **p-value** | **AOR (95% CI)*** | **p-value** | **AOR (95% CI)*** | **p-value** | **AOR (95% CI)*** | **p-value** | **AOR (95% CI)*** | **p-value** | **AOR (95% CI)*** | **p-value** |
| Age group  18 to 44  45 or older | Ref  0.85 (0.73- 0.98) | Ref  0.029 | Ref  1.23 (1.05- 1.43) | Ref  0.008 | Ref  0.92 (0.79- 1.01) | Ref  0.26 | Ref  0.83 (0.71- 0.96) | Ref  0.012 | Ref  0.94 (0.81- 1.09) | Ref  0.38 | Ref  1.41 (1.21- 1.64) | Ref  <0.001 | Ref  0.91 (0.78- 1.08) | Ref  0.29 |
| Menarche  Normal  Early  Late | Ref  1.36 (0.8- 2.31)  1.02 (0.87- 1.20) | Ref  0.25  0.80 | Ref  0.96 (0.58- 1.58)  1.12 (0.95- 1.32) | Ref  0.86  0.19 | Ref  1.32 (0.78- 2.22)  0.93 (0.79- 1.09) | Ref  0.30  0.36 | Ref  1.23 (0.74- 2.05)  1.14 (0.97- 1.35) | Ref  0.43  0.11 | Ref  1.29 (0.78- 2.13)  1.10 (0.94- 1.30) | Ref  0.32  0.21 | Ref  1.24 (0.75- 2.06)  0.97 (0.82- 1.15) | Ref  0.40  0.74 | Ref  1.14 (0.68- 1.91)  1.08 (0.91- 1.28) | Ref  0.62  0.39 |
| Educational level  Secondary or below  Post-secondary | Ref  1.40 (1.23- 1.59) | Ref  <0.001 | Ref  1.14 (1.01- 1.30) | Ref  0.037 | Ref  1.26 (1.11- 1.43) | Ref  <0.001 | Ref  1.17 (1.03- 1.32) | Ref  0.017 | Ref  1.23 (1.08- 1.39) | Ref  0.001 | Ref  1.20 (1.05- 1.37) | Ref  0.006 | Ref  1.44 (1.26- 1.65) | Ref  <0.001 |
| Occupation  Unemployed  Employed  Retired  Student | Ref  0.93 (0.79- 1.10)  0.77 (0.42- 1.41)  1.22 (0.97- 1.54) | Ref  0.42  0.40  0.09 | Ref  0.92 (0.79- 1.09)  0.68 (0.37- 1.23)  0.96 (0.77- 1.20) | Ref  0.35  0.20  0.71 | Ref  0.87 (0.74- 1.02)  0.69 (0.38- 1.26)  0.95 (0.76- 1.19) | Ref  0.09  0.23  0.64 | Ref  0.99 (0.84- 1.16)  0.75 (0.41- 1.38)  0.88 (0.70- 1.09) | Ref  0.89  0.36  0.24 | Ref  1.00 (0.85- 1.18)  1.02 (0.56- 1.87)  0.89 (0.72- 1.11) | Ref  0.99  0.94  0.31 | Ref  1.05 (0.90- 1.26)  0.53 (0.26- 1.05)  1.07 (0.85- 1.36) | Ref  0.46  0.07  0.56 | Ref  1.28 (1.08- 1.51)  0.57 (0.26- 1.22)  1.32 (1.05- 1.67) | Ref  0.005  0.15  0.017 |
| Monthly income  < 1450 NIS  ≥ 1450 NIS | Ref  1.21 (1.03- 1.44) | Ref  0.023 | Ref  1.11 (0.94- 1.31) | Ref  0.24 | Ref  1.26 (1.07- 1.48) | Ref  0.006 | Ref  1.31 (1.11- 1.54) | Ref  0.002 | Ref  1.20 (1.02- 1.41) | Ref  0.031 | Ref  1.22 (1.03- 1.45) | Ref  0.022 | Ref  1.07 (0.90- 1.28) | Ref  0.44 |
| Residency  Gaza Strip  WBJ | Ref  0.78 (0.66- 0.92) | Ref  0.003 | Ref  0.72 (0.61- 0.84) | Ref  <0.001 | Ref  1.29 (1.10- 1.51) | Ref  0.002 | Ref  0.60 (0.51- 0.71) | Ref  <0.001 | Ref  0.72 (0.61- 0.84) | Ref  <0.001 | Ref  0.51 (0.43- 0.60) | Ref  <0.001 | Ref  0.65 (0.55- 0.77) | Ref  <0.001 |
| Having a chronic disease  No  Yes | Ref  0.94 (0.81- 1.09) | Ref  0.43 | Ref  0.99 (0.85- 1.16) | Ref  0.93 | Ref  1.05 (0.90- 1.22) | Ref  0.54 | Ref  1.06 (0.91- 1.23) | Ref  0.48 | Ref  0.99 (0.85- 1.15) | Ref  0.92 | Ref  1.00 (0.85- 1.17) | Ref  0.99 | Ref  1.01 (0.85- 1.19) | Ref  0.95 |
| Knowing someone with cancer  No  Yes | Ref  1.18 (1.06- 1.32) | Ref  0.003 | Ref  1.18 (1.06- 1.33) | Ref  0.003 | Ref  1.43 (1.28- 1.60) | Ref  <0.001 | Ref  1.23 (1.10- 1.37) | Ref  <0.001 | Ref  1.42 (1.23- 1.58) | Ref  <0.001 | Ref  1.11 (1.01- 1.25) | Ref  0.047 | Ref  1.23 (1.09- 1.38) | Ref  0.001 |
| Marital status  Single  Married  Divorced  Widowed | Ref  1.13 (0.95- 1.33)  0.80 (0.51- 1.25)  1.39 (0.93- 2.10) | Ref  0.16  0.32  0.11 | Ref  1.21 (1.03- 1.43)  0.85 (0.54- 1.33)  0.89 (0.59- 1.33) | Ref  0.023  0.47  0.56 | Ref  1.13 (0.96- 1.34)  1.48 (0.94- 2.34)  1.54 (1.02- 2.33) | Ref  0.14  0.09  0.040 | Ref  1.24 (1.05- 1.46)  1.14 (0.73- 1.78)  1.31 (0.87- 1.97) | Ref  0.010  0.59  0.12 | Ref  1.09 (0.92- 1.28)  0.98 (0.63- 1.53)  1.21 (0.81- 1.80) | Ref  0.32  0.93  0.36 | Ref  1.31 (1.10- 1.56)  0.86 (0.53- 1.41)  1.20 (0.79- 1.82) | Ref  0.003  0.56  0.40 | Ref  1.19 (1.00- 1.42)  0.75 (0.46- 1.24)  1.03 (0.65- 1.61) | Ref  0.049  0.27  0.91 |
| Site of data collection  Public spaces  Hospitals  PHCs | Ref  0.79 (0.68- 0.91)  0.59 (0.52- 0.68) | Ref  0.001  <0.001 | Ref  1.49 (1.28- 1.72)  0.80 (0.70- 0.92) | Ref  <0.001  0.002 | Ref  1.31 (1.13- 1.51)  0.98 (0.85- 1.13) | Ref  <0.001  0.78 | Ref  1.49 (1.29- 1.73)  0.81 (0.71- 0.94) | Ref  <0.001  0.004 | Ref  1.03 (0.89- 1.19)  0.68 (0.59- 0.78) | Ref  0.71  <0.001 | Ref  1.00 (0.86- 1.16)  0.75 (0.64- 0.86) | Ref  0.98  <0.001 | Ref  0.47 (0.40- 0.54)  0.31 (0.27- 0.36) | Ref  <0.001  <0.001 |

Supplementary table 2: Multivariable logistic regression analyzing the association between recognizing the non-modifiable risk factors of ovarian cancer and participant characteristics.

AOR= adjusted odds ratio, CI= confidence interval, WBJ= West Bank and Jerusalem, PHCs= Primary healthcare centers.
* Adjusted for age-group, menarche, educational level, occupation, monthly income, residency, having a chronic disease, knowing someone with cancer, and site of data collection.

| Characteristic | Breastfeeding | | Undergoing prophylactic bilateral oophorectomy | | Using the pill for a long time | |
| --- | --- | --- | --- | --- | --- | --- |
|  | **AOR (95% CI)*** | **p-value** | **AOR (95% CI)*** | **p-value** | **AOR (95% CI)*** | **p-value** |
| Age group  18 to 44  45 or older | Ref  1.19 (0.92- 1.52) | Ref  0.19 | Ref  0.89 (0.76- 1.03) | Ref  0.13 | Ref  0.94 (0.78- 1.15) | Ref  0.55 |
| Menarche  Normal  Early  Late | Ref  1.78 (0.63- 5.01)  1.05 (0.80- 1.37) | Ref  0.27  0.74 | Ref  0.84 (0.51- 1.40)  1.08 (0.91- 1.27) | Ref  0.51  0.37 | Ref  0.50 (0.22- 1.17)  1.12 (0.91- 1.38) | Ref  0.11  0.28 |
| Educational level  Secondary or below  Post-secondary | Ref  0.95 (0.78- 1.16) | Ref  0.62 | Ref  1.24 (1.09- 1.41) | Ref  0.001 | Ref  0.92 (0.78- 1.08) | Ref  0.30 |
| Occupation  Unemployed/housewife  Employed  Retired  Student | Ref  0.47 (0.38- 0.60)  0.42 (0.20- 0.88)  1.12 (0.80- 1.56) | Ref  <0.001  0.023  0.51 | Ref  0.76 (0.65- 0.90)  0.84 (0.45- 1.56)  0.95 (0.76- 1.19) | Ref  0.001  0.58  0.67 | Ref  0.82 (0.66- 1.02)  0.47 (0.17- 1.34)  0.61 (0.45- 0.84) | Ref  0.08  0.16  0.002 |
| Monthly income  < 1450 NIS  ≥ 1450 NIS | Ref  0.83 (0.63- 1.10) | Ref  0.19 | Ref  1.14 (0.96- 1.34) | Ref  0.14 | Ref  0.83 (0.67- 1.03) | Ref  0.09 |
| Residency  Gaza Strip  WBJ | Ref  0.67 (0.52- 0.88) | Ref  0.004 | Ref  1.27 (1.08- 1.50) | Ref  0.004 | Ref  1.07 (0.86- 1.32) | Ref  0.56 |
| Having a chronic disease  No  Yes | Ref  1.01 (0.78- 1.30) | Ref  0.93 | Ref  0.93 (0.79- 1.08) | Ref  0.34 | Ref  0.92 (0.75- 1.12) | Ref  0.42 |
| Knowing someone with cancer  No  Yes | Ref  1.78 (1.48- 2.13) | Ref  <0.001 | Ref  1.35 (1.20- 1.51) | Ref  <0.001 | Ref  0.83 (0.72- 0.96) | Ref  0.013 |
| Marital status  Single  Married  Divorced  Widowed | Ref  1.90 (1.52- 2.39)  1.13 (0.62- 2.06)  1.20 (0.65- 2.21) | Ref  <0.001  0.68  0.57 | Ref  1.09 (0.92- 1.29)  0.64 (0.39- 1.06)  1.00 (0.66- 1.51) | Ref  0.30  0.08  0.97 | Ref  0.99 (0.79- 1.23)  1.03 (0.57- 1.86)  1.04 (0.61- 1.76) | Ref  0.93  0.92  0.89 |
| Site of data collection  Public spaces  Hospitals  Primary healthcare centers | Ref  1.01 (0.79- 1.30)  0.55 (0.45- 0.69) | Ref  0.92  <0.001 | Ref  0.94 (0.81- 1.09)  0.70 (0.61- 0.81) | Ref  0.40  <0.001 | Ref  1.17 (0.97- 1.41)  0.90 (0.75- 1.09) | Ref  0.10  0.27 |

AOR= adjusted odds ratio, CI= confidence interval, WBJ= West Bank and Jerusalem.
* Adjusted for age-group, menarche, educational level, occupation, monthly income, residency, having a chronic disease, knowing someone with cancer, and site of data collection

Supplementary table 3: Multivariable logistic regression analyzing the association between recognizing the protective factors of ovarian cancer and participant characteristics.

| Characteristic | Being a smoker | | Using hormone replacement therapy | | Being overweight | | Using talcum powder in the genital area | | Having IVF treatment | |
| --- | --- | --- | --- | --- | --- | --- | --- | --- | --- | --- |
|  | **COR (95% CI)** | **p-value** | **COR (95% CI)** | **p-value** | **COR (95% CI)** | **p-value** | **COR (95% CI)** | **p-value** | **COR (95% CI)** | **p-value** |
| Age group  18to 44  45 or older | Ref  1.09 (0.94- 1.26) | Ref  0.24 | Ref  1.08 (0.95- 1.23) | Ref  0.24 | Ref  1.06 (0.93- 1.20) | Ref  0.37 | Ref  0.93 (0.82- 1.05) | Ref  0.25 | Ref  0.91 (0.79- 1.05) | Ref  0.19 |
| Menarche  Normal  Early  Late | Ref  1.17 (0.66- 2.10)  1.16 (0.96- 1.40) | Ref  0.59  0.12 | Ref  1.15 (0.70- 1.89)  0.92 (0.79- 1.08) | Ref  0.59  0.33 | Ref  0.99 (0.61- 1.63)  1.20 (1.02- 1.41) | Ref  0.98  0.027 | Ref  0.96 (0.59- 1.58)  0.91 (0.77- 1.07) | Ref  0.88  0.26 | Ref  1.18 (0.71- 1.99)  1.12 (0.94- 1.33) | Ref  0.52  0.21 |
| Educational level  Secondary or below  Post-secondary | Ref  0.71 (0.62- 0.80) | Ref  <0.001 | Ref  1.35 (1.21- 1.50) | Ref  <0.001 | Ref  0.94 (0.85- 1.05) | Ref  0.30 | Ref  1.12 (1.00 -1.24) | Ref  0.046 | Ref  1.20 (1.07- 1.35) | Ref  0.002 |
| Occupation  Unemployed/housewife  Employed  Retired  Student | Ref  0.58 (0.50- 0.67)  0.69 (0.37- 1.30)  0.73 (0.60- 0.88) | Ref  <0.001  0.25  0.001 | Ref  1.06 (0.92- 1.21)  1.65 (0.89- 3.06)  0.88 (0.74- 1.04) | Ref  0.41  0.11  0.14 | Ref  0.92 (0.80- 1.05)  0.78 (0.44- 1.41)  0.79 (0.66- 0.94) | Ref  0.23  0.41  0.008 | Ref  0.92 (0.80- 1.05)  0.84 (0.47- 1.52)  0.85 (0.71- 1.01) | Ref  0.23  0.57  0.07 | Ref  1.08 (0.94- 1.26)  0.72 (0.37- 1.42)  1.21 (1.01- 1.46) | Ref  0.27  0.34  0.040 |
| Monthly income  < 1450 NIS  ≥ 1450 NIS | Ref  0.75 (0.66- 0.85) | Ref  <0.001 | Ref  1.29 (1.16- 1.44) | Ref  <0.001 | Ref  0.81 (0.72- 0.90) | Ref  <0.001 | Ref  1.16 (1.04- 1.30) | Ref  0.009 | Ref  0.80 (0.71- 0.90) | Ref  <0.001 |
| Residency  Gaza Strip  WBJ | Ref  0.72 (0.63- 0.81) | Ref  <0.001 | Ref  1.04 (0.93- 1.16) | Ref  0.46 | Ref  0.78 (0.70- 0.86) | Ref  <0.001 | Ref  1.07 (0.96- 1.20) | Ref  0.21 | Ref  0.74 (0.66- 0.83) | Ref  <0.001 |
| Having a chronic disease  No  Yes | Ref  1.09 (0.93- 1.27) | Ref  0.27 | Ref  1.04 (0.91- 1.19) | Ref  0.54 | Ref  1.06 (0.92- 1.21) | Ref  0.43 | Ref  1.06 (0.93- 1.22) | Ref  0.36 | Ref  0.86 (0.75- 0.99) | Ref  0.048 |
| Knowing someone with cancer  No  Yes | Ref  1.37 (1.21- 1.55) | Ref  <0.001 | Ref  1.07 (0.96- 1.19) | Ref  0.20 | Ref  1.06 (0.95- 1.18) | Ref  0.27 | Ref  1.08 (0.97- 1.21) | Ref  0.14 | Ref  1.04 (0.93- 1.17) | Ref  0.48 |
| Marital status  Single  Married  Divorced  Widowed | Ref  1.57 (1.36- 1.80)  2.20 (1.26- 3.83)  2.38 (1.46- 3.86) | Ref  <0.001  0.005  <0.001 | Ref  1.24 (1.09- 1.41)  0.82 (0.53- 1.27)  0.98 (0.67- 1.41) | Ref  0.001  0.37  0.90 | Ref  1.29 (1.14- 1.47)  1.41 (0.91- 2.17)  1.19 (0.82- 1.73) | Ref  <0.0010.12  0.36 | Ref  1.25 (1.10- 1.43)  1.01 (0.65- 1.57)  1.23 (0.85- 1.79) | Ref  0.001  0.97  0.27 | Ref  0.92 (0.80- 1.06)  0.63 (0.37- 1.04)  0.99 (0.66- 1.47) | Ref  0.24  0.07  0.95 |
| Site of data collection  Public spaces  Hospitals  Primary healthcare centers | Ref  1.61 (1.37- 1.89)  0.91 (0.79- 1.06) | Ref  <0.001  0.22 | Ref  1.10 (0.96- 1.26)  0.89 (0.78- 1.02) | Ref  0.19  0.08 | Ref  1.30 (1.14- 1.49)  1.21 (1.06- 1.38) | Ref  <0.0010.004 | Ref  1.16 (1.02- 1.33)  1.11 (0.97- 1.26) | Ref  0.029  0.13 | Ref  0.69 (0.60- 0.80)  0.74 (0.64- 0.85) | Ref  <0.001  <0.001 |

Supplementary table 4: Bivariable logistic regression analyzing the association between recognizing the modifiable risk factors of ovarian cancer and participant characteristics.

COR= crude odds ratio, CI= confidence interval, WBJ= West Bank and Jerusalem, IVF= in vitro fertilization.

| Characteristic | Having ovarian cysts | | Being over 50 years old | | Having a close relative with ovarian cancer | | Having gone through the menopause | | Having a history of breast cancer | | Having no children | | Having endometriosis | |
| --- | --- | --- | --- | --- | --- | --- | --- | --- | --- | --- | --- | --- | --- | --- |
|  | **COR (95% CI)** | **p-value** | **COR (95% CI)** | **p-value** | **COR (95% CI)** | **p-value** | **COR (95% CI)** | **p-value** | **COR (95% CI)** | **p-value** | **COR (95% CI)** | **p-value** | **COR (95% CI)** | **p-value** |
| Age group  18to 44  45 or older | Ref  0.77 (0.68- 0.87) | Ref  <0.001 | Ref  1.22 (1.07- 1.38) | Ref  0.002 | Ref  1.03 (0.91- 1.17) | Ref  0.60 | Ref  0.89 (0.78- 1.00) | Ref  0.06 | Ref  0.95 (0.84- 1.07) | Ref  0.40 | Ref  1.30 (1.14- 1.48) | Ref  <0.001 | Ref  0.79 (0.69- 0.90) | Ref  <0.001 |
| Menarche  Normal  Early  Late | Ref  1.53 (0.91- 2.58)  1.01 (0.86- 1.18) | Ref  0.11  0.93 | Ref  1.02 (0.62- 1.67)  1.16 (0.99- 1.37) | Ref  0.93  0.07 | Ref  1.48 (0.88- 2.46)  0.90 (0.76- 1.05) | Ref  0.14  0.19 | Ref  1.29 (0.78- 2.12)  1.18 (1.00- 1.38) | Ref  0.32  0.047 | Ref  1.45 (0.88- 2.38)  1.12 (0.95- 1.31) | Ref  0.15  0.18 | Ref  1.29 (0.79- 2.12)  1.01 (0.86- 1.20) | Ref  0.31  0.89 | Ref  1.35 (0.82- 2.22)  1.06 (0.89- 1.25) | Ref  0.24  0.52 |
| Educational level  Secondary or below  Post-secondary | Ref  1.38 (1.24- 1.54) | Ref  <0.001 | Ref  0.95 (0.85- 1.06) | Ref  0.34 | Ref  1.13 (1.01- 1.26) | Ref  0.028 | Ref  1.03 (0.93- 1.15) | Ref  0.53 | Ref  1.15 (1.03- 1.28) | Ref  0.012 | Ref  1.06 (0.95- 1.19) | Ref  0.29 | Ref  1.52 (1.36- 1.70) | Ref  <0.001 |
| Occupation  Unemployed  Employed  Retired  Student | Ref  1.09 (0.95- 1.25)  0.73 (0.41- 1.31)  1.47 (1.23- 1.76) | Ref  0.24  0.29  <0.001 | Ref  0.82 (0.72- 0.94)  0.72 (0.40- 1.27)  0.74 (0.62- 0.88) | Ref  0.005  0.26  0.001 | Ref  1.04 (0.91- 1.19)  0.85 (0.48- 1.52)  0.96 (0.81- 1.14) | Ref  0.58  0.59  0.65 | Ref  0.90 (0.78- 1.03)  0.64 (0.36- 1.14)  0.72 (0.61- 0.86) | Ref  0.12  0.13  <0.001 | Ref  1.03 (0.90- 1.17)  0.96 (0.54- 1.71)  0.92 (0.77- 1.10) | Ref  0.72  0.90  0.35 | Ref  0.94 (0.82- 1.09)  0.60 (0.31- 1.15)  0.80 (0.67- 0.97) | Ref  0.42  0.12  0.021 | Ref  1.42 (1.23- 1.63)  0.51 (0.24- 1.05)  1.71 (1.44- 2.04) | Ref  <0.001  0.07  <0.001 |
| Monthly income  < 1450 NIS  ≥ 1450 NIS | Ref  1.12 (0.99- 1.25) | Ref  0.054 | Ref  0.89 (0.79- 0.99) | Ref  0.030 | Ref  1.50 (1.34- 1.67) | Ref  <0.001 | Ref  0.91 (0.82- 1.02) | Ref  0.10 | Ref  1.00 (0.90- 1.12) | Ref  0.97 | Ref  0.80 (0.72- 0.90) | Ref  <0.001 | Ref  1.00 (0.89- 1.12) | Ref  0.99 |
| Residency  Gaza Strip  WBJ | Ref  0.93 (0.84- 1.04) | Ref  0.23 | Ref  0.80 (0.72- 0.89) | Ref  <0.001 | Ref  1.51 (1.35- 1.68) | Ref  <0.001 | Ref  0.75 (0.67- 0.83) | Ref  <0.001 | Ref  0.86 (0.77- 0.96) | Ref  0.007 | Ref  0.63 (0.56- 0.70) | Ref  <0.001 | Ref  0.81 (0.72- 0.91) | Ref  <0.001 |
| Having a chronic disease  No  Yes | Ref  0.80 (0.70- 1.04) | Ref  0.001 | Ref  1.10 (0.96- 1.26) | Ref  0.15 | Ref  1.11 (0.97- 1.27) | Ref  0.12 | Ref  1.00 (0.88- 1.14) | Ref  0.98 | Ref  0.96 (0.84- 1.09) | Ref  0.50 | Ref  1.08 (0.94- 1.23) | Ref  0.31 | Ref  0.79 (0.69- 0.91) | Ref  0.001 |
| Knowing someone with cancer  No  Yes | Ref  1.21 (1.08- 1.34) | Ref  0.001 | Ref  1.21 (1.08- 1.34) | Ref  0.001 | Ref  1.45 (1.30- 1.61) | Ref  <0.001 | Ref  1.22 (1.10- 1.36) | Ref  <0.001 | Ref  1.44 (1.29- 1.60) | Ref  <0.001 | Ref  1.14 (1.02- 1.27) | Ref  0.025 | Ref  1.31 (1.17- 1.47) | Ref  <0.001 |
| Marital status  Single  Married  Divorced  Widowed | Ref  0.85 (0.75- 0.97)  0.64 (0.42- 0.99)  0.87 (0.60- 1.27) | Ref  0.018  0.043  0.48 | Ref  1.35 (1.19- 1.53)  0.88 (0.57- 1.35)  1.09 (0.75- 1.58) | Ref  <0.001  0.56  0.64 | Ref  1.08 (0.95- 1.23)  1.33 (0.85- 2.06)  1.47 (1.00- 2.15) | Ref  0.22  0.21  0.049 | Ref  1.34 (1.18- 1.53)  1.09 (0.71- 1.69)  1.21 (0.84- 1.76) | Ref  <0.001  0.68  0.31 | Ref  1.07 (0.94- 1.21)  0.97 (0.63- 1.49)  1.11 (0.77- 1.61) | Ref  0.33  0.88  0.57 | Ref  1.34 (1.17- 1.54)  0.90 (0.56- 1.45)  1.35 (0.92- 1.98) | Ref  <0.001  0.65  0.13 | Ref  0.76 (0.66- 0.86)  0.67 (0.42- 1.07)  0.63 (0.42- 0.94) | Ref  <0.001  0.09  0.023 |
| Site of data collection  Public spaces  Hospitals  PHCs | Ref  0.71 (0.62- 0.81)  0.57 (0.50- 0.66) | Ref  <0.001  <0.001 | Ref  1.54 (1.34- 1.77)  0.86 (0.75- 0.98) | Ref  <0.001  0.020 | Ref  1.23 (1.08- 1.41)  0.88 (0.78- 1.01) | Ref  0.003  0.06 | Ref  1.50 (1.31- 1.72)  0.87 (0.77- 0.99) | Ref  <0.001  0.038 | Ref  0.99 (0.86- 1.13)  0.68 (0.60- 0.77) | Ref  0.84  <0.001 | Ref  1.03 (0.89- 1.18)  0.82 (0.72-0.94) | Ref  0.71  0.004 | Ref  0.42 (0.37- 0.49)  0.32 (0.28- 0.37) | Ref  <0.001  <0.001 |

Supplementary table 5: Bivariable logistic regression analyzing the association between recognizing the non-modifiable risk factors of ovarian cancer and participant characteristics.

AOR= adjusted odds ratio, CI= confidence interval, WBJ= West Bank and Jerusalem, PHCs= Primary healthcare centers.
* Adjusted for age-group, menarche, educational level, occupation, monthly income, residency, having a chronic disease, knowing someone with cancer, and site of data collection.

| Characteristic | Breastfeeding | | Undergoing prophylactic bilateral oophorectomy | | Using the pill for a long time | |
| --- | --- | --- | --- | --- | --- | --- |
|  | **COR (95% CI)** | **p-value** | **COR (95% CI)** | **p-value** | **COR (95% CI)** | **p-value** |
| Age group  18 to 44  45 or older | Ref  1.25 (1.02- 1.53) | Ref  0.035 | Ref  0.89 (0.78- 1.02) | Ref  0.08 | Ref  0.97 (0.82- 1.15) | Ref  0.76 |
| Menarche  Normal  Early  Late | Ref  2.11 (0.76- 5.82)  1.20 (0.93- 1.56) | Ref  0.15  0.17 | Ref  0.97 (0.59- 1.61)  1.05 (0.89- 1.24) | Ref  0.91  0.53 | Ref  0.50 (0.21- 1.16)  1.17 (0.96- 1.44) | Ref  0.10  0.13 |
| Educational level  Secondary or below  Post-secondary | Ref  0.53 (0.45-0.62) | Ref  <0.001 | Ref  1.13 (1.01- 1.26) | Ref  0.027 | Ref  0.81 (0.71- 0.94) | Ref  0.005 |
| Occupation  Unemployed/housewife  Employed  Retired  Student | Ref  0.31 (0.26- 0.37)  0.30 (0.15- 0.59)  0.62 (0.48- 0.81) | Ref  <0.001  0.001  0.001 | Ref  0.91 (0.79- 1.04)  0.87 (0.48- 1.58)  1.13 (0.95- 1.35) | Ref  0.17  0.65  0.16 | Ref  0.75 (0.63- 0.91)  0.40 (0.14- 1.21)  0.59 (0.46- 0.77) | Ref  0.003  0.08  <0.001 |
| Monthly income  < 1450 NIS  ≥ 1450 NIS | Ref  0.53 (0.44- 0.64) | Ref  <0.001 | Ref  1.36 (1.22- 1.53) | Ref  <0.001 | Ref  0.79 (0.69- 0.91) | Ref  0.001 |
| Residency  Gaza Strip  WBJ | Ref  0.52 (0.44- 0.63) | Ref  <0.001 | Ref  1.37 (1.22- 1.53) | Ref  <0.001 | Ref  0.87 (0.76- 1.01) | Ref  0.06 |
| Having a chronic disease  No  Yes | Ref  1.23 (0.99- 1.52) | Ref  0.06 | Ref  0.91 (0.80- 1.04) | Ref  0.18 | Ref  0.96 (0.81- 1.15) | Ref  0.68 |
| Knowing someone with cancer  No  Yes | Ref  2.01 (1.69- 2.39) | Ref  <0.001 | Ref  1.38 (1.24- 1.54) | Ref  <0.001 | Ref  0.84 (0.73-0.97) | Ref  0.020 |
| Marital status  Single  Married  Divorced  Widowed | Ref  2.29 (1.92- 2.73)  1.07 (0.61- 1.87)  1.71 (0.98- 2.98) | Ref  <0.001  0.82  0.06 | Ref  0.97 (0.85- 1.10)  0.56 (0.35- 0.91)  0.83 (0.56- 1.22) | Ref  0.65  0.018  0.35 | Ref  1.25 (1.05- 1.50)  1.18 (0.66- 2.10)  1.25 (0.77- 2.04) | Ref  0.012  0.57  0.37 |
| Site of data collection  Public spaces  Hospitals  Primary healthcare centers | Ref  1.21 (0.96- 1.52)  0.62 (0.51- 0.76) | Ref  0.11  <0.001 | Ref  0.91 (0.79- 1.04)  0.66 (0.58- 0.75) | Ref  0.16  <0.001 | Ref  1.333 (1.11- 1.59)  1.02 (0.86- 1.22) | Ref  0.002  0.80 |

Supplementary table 6: Bivariable logistic regression analyzing the association between recognizing the protective factors of ovarian cancer and participant characteristics.

COR= crude odds ratio, CI= confidence interval, WBJ= West Bank and Jerusalem.
